# A H_2_O_2_-Responsive Boron Dipyrromethene-Based Photosensitizer for Imaging-Guided Photodynamic Therapy

**DOI:** 10.3390/molecules24010032

**Published:** 2018-12-21

**Authors:** Zhi-Wei Wang, Dan Su, Xiao-Qiang Li, Jing-Jing Cao, De-Chao Yang, Jian-Yong Liu

**Affiliations:** State Key Laboratory of Photocatalysis on Energy and Environment & National & Local Joint Biomedical Engineering Research Center on Photodynamic Technologies, College of Chemistry, Fuzhou University, Fuzhou 350108, China; m15139226887_2@163.com (Z.-W.W.); N171320109@fzu.edu.cn (D.S.); n161320100@fzu.edu.cn (X.-Q.L.); cjj0528dch@163.com (J.-J.C.); yangdechao7258@163.com (D.-C.Y.)

**Keywords:** photodynamic therapy, activatable photosensitizers, H_2_O_2_-responsive, photoinduced electron transfer (PET)

## Abstract

In this study, we demonstrate a novel H_2_O_2_ activatable photosensitizer (compound **7**) which contains a diiodo distyryl boron dipyrromethene (BODIPY) core and an arylboronate group that quenches the excited state of the BODIPY dye by photoinduced electron transfer (PET). The BODIPY-based photosensitizer is highly soluble and remains nonaggregated in dimethyl sulfoxide (DMSO) as shown by the intense and sharp Q-band absorption (707 nm). As expected, compound **7** exhibits negligible fluorescence emission and singlet oxygen generation efficiency. However, upon interaction with H_2_O_2_, both the fluorescence emission and singlet oxygen production of the photosensitizer can be restored in phosphate buffered saline (PBS) solution and PBS buffer solution containing 20% DMSO as a result of the cleavage of the arylboronate group. Due to the higher concentration of H_2_O_2_ in cancer cells, compound **7** even with low concentration is particularly sensitive to human cervical carcinoma (HeLa) cells (IC_50_ = 0.95 μM) but hardly damage human embryonic lung fibroblast (HELF) cells. The results above suggest that this novel BODIPY derivative is a promising candidate for fluorescence imaging-guided photodynamic cancer therapy.

## 1. Introduction

Photodynamic therapy (PDT) has received great attention for their advantages such as minimal invasiveness, high selectivity and low immunogenicity [1,2]. The method involves three elements: near-infrared light, molecular oxygen and photosensitizers, which can generate cytotoxic reactive oxygen species (ROS) in tumors region to induce cancer cells apoptosis and necrosis [3]. However, a drawback of the typical PDT approach is that the therapeutic effect subjects to the depth and range of light exposure [4]. Otherwise, some patients must remain in dark for long periods of time avoiding photo-allergic reactions [5,6], for instance, rashes or blisters. Also, the selectivity of typical photodynamic therapy is still not as high as expected, and the therapeutic effect is not ideal yet [7,8].

Recently, a new PDT technique that utilizes tumor microenvironment stimuli to activate photosensitizers selectively has been reported [9,10,11]. And this kind of photosensitizers, called activatable photosensitizers (aPS), exists in a passive state in normal tissues even upon exposure to treatment window light [11]. When going into tumor microenvironments, the sensitive linkers between the photosensitizers and quenchers are cut off, thus the photosensitizers restore their activities. And then exposed to the near-infrared light, they can interact with molecular oxygen to generate cytotoxic reactive oxygen species (ROS), like singlet oxygen (^1^O_2_) to induce cancer cells apoptosis and necrosis [12]. There are many ways to break chemical bonds between photosensitizers and quenchers since tumor tissues have unique microenvironments [13], for example, low pH [14,15,16,17], high expressed enzymes [18,19,20], high concentration of intracellular glutathione (GSH) (ca. 10 mM) [21,22,23] and high reactive oxygen species level (including H_2_O_2_) [24,25,26,27].

**Scheme 1:**
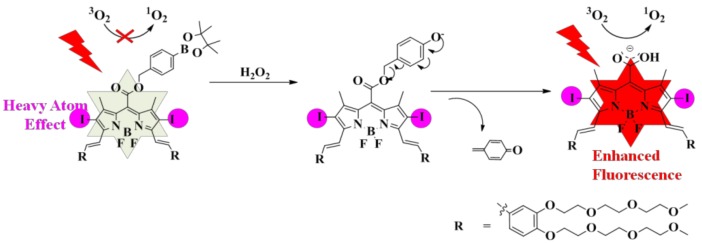
Schematic illustration of proposed activation mechanism.

On the other hand, considering the higher concentration of H_2_O_2_ in cancer cells, a large variety of H_2_O_2_-responsive fluorescent dyes have been widely employed in previous research [28], mainly based on fluorescence resonance energy transfer (FRET) [29], intramolecular charge transfer (ICT) [30] and photoinduced electron transfer (PET) [31] mechanisms. And the probes are constructed with boronate esters [32], aromatic thioethers [33] or oxalate esters [34], all of which react to H_2_O_2_. However, to the best of our knowledge, H_2_O_2_ activatable photosensitizers remain very rare [35]. Indeed, a large body of evidence indicates that H_2_O_2_ and other ROS play important roles in health and physiological signaling pathways [36]. Herein, we synthesize a H_2_O_2_-responsive photosensitizer for near infrared (NIR) fluorescence imaging-guided photodynamic therapy. As depicted in Scheme 1, in the presence of H_2_O_2_, the arylboronate group is broken, the *meso*-ester-substituted dye converts to highly labile intermediates, followed by the linker self-immolation to form *meso*-carboxylate-substituted BODIPY with strong fluorescence emission [37]. And when exposed to the near-infrared light, it would generate singlet oxygen to kill the cancer cells. These ideal features of tumor microenvironment-activated ability, effective therapeutic effect and NIR fluorescence imaging make this kind of PS a promising anticancer agent for selective photodynamic therapy.

## 2. Results and Discussion

### 2.1. Molecular Design and Chemical Synthesis

**Figure 1:**
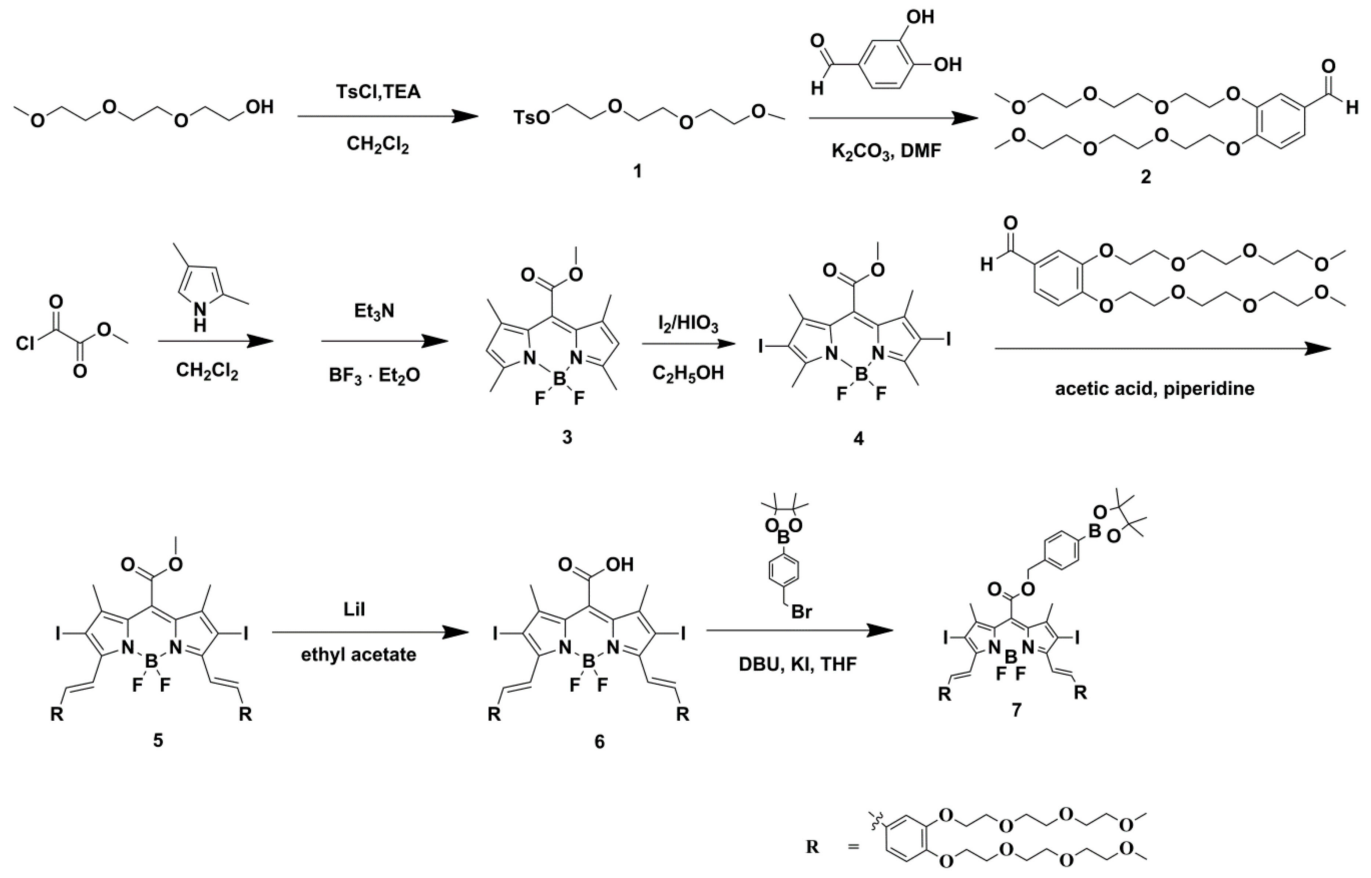
Synthesis of the H_2_O_2_-responsive photosensitizer.

Here, a new type of H_2_O_2_-activatable photosensitizer, derived from the design of molecular fluorescent probes in previous investigation [30], have been designed for fluorescence imaging-guided photodynamic therapy. It is well known that the core structure, a BODIPY dye, without any modifications is known to be very poor photosensitizer. Therefore, we introduce iodine atoms incorporated into the 2 and 6 positions of the BODIPY core, which can facilitate intersystem crossing to promote the generation of singlet oxygen [38]. Also, in order to improve the amphiphilicity and biocompatibility, four oligoethyleneglycol moieties are added into the BODIPY core [39]. Furthermore, the BODIPY dye bearing an electron withdrawing arylboronate group which quenches the excited state of the photosensitizer (PS) by photoinduced electron transfer (PET) (Scheme 1), can remain in a quenched state in normal tissue so as not to damage them. Figure 1 shows the detailed synthetic route for the H_2_O_2_-activatable photosensitizer **7**. Firstly, compound **3** was prepared by condensation of 2,4-dimethyl pyrrole with methyl chlorooxoacetate, followed by complexation with BF_3_·Et_2_O in dichloromethane. This compound then underwent electrophilic substitution with I_2_ and HIO_3_ to give iodo-BODIPY **4**, which was treated with aldehyde **2** to afford **5**. Compound **5** was hydrolyzed to **6** in the presence of LiI. Finally, compound **7** was obtained by treatment of compound **6** with 4-(Bromomethyl)benzeneboronic acid pinacol ester.

### 2.2. Photochemical and Photophysical Properties

**Figure 2:**
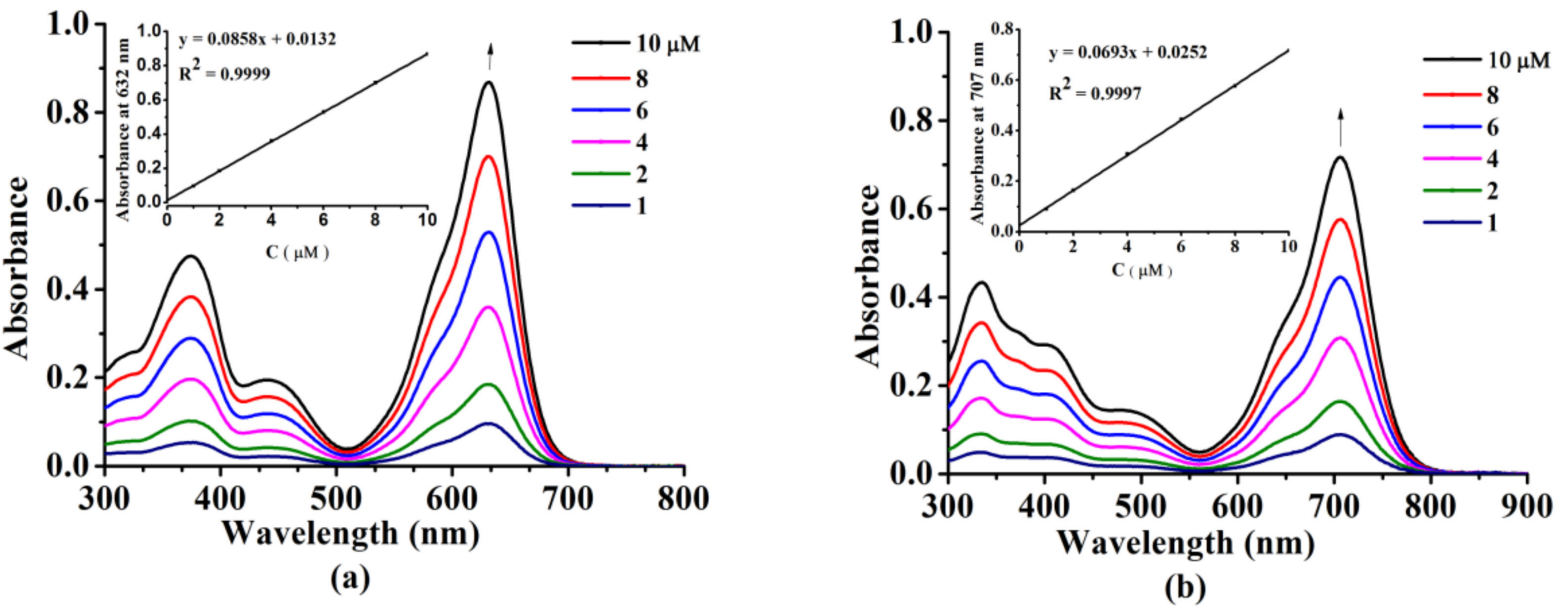
Electronic absorption spectra of compounds **6** (**a**) and **7** (**b**) at different concentrations in DMSO. The inset plots the Q-band absorbance versus the concentration of compounds.

The electronic absorption spectrum in DMSO shows that compound **7** is a typical non-aggregated BODIPY derivative with a strong Q-band absorption at 707 nm, whose position shows a heavy red-shift (75 nm) compared to compound **6** (Figure 2, Table 1). Upon excitation at 610 nm, compound **7** shows a very weak fluorescence emission at 755 nm with a fluorescence quantum yield (Φ_F_) of 0.067, which is significantly weaker than that of the compound **6** (Φ_F_ = 0.17) (Table 1). The results clearly indicate that the fluorescence of compound **7** is largely quenched by the arylboronate group by the photoinduced electron transfer (PET).

**Figure 3:**
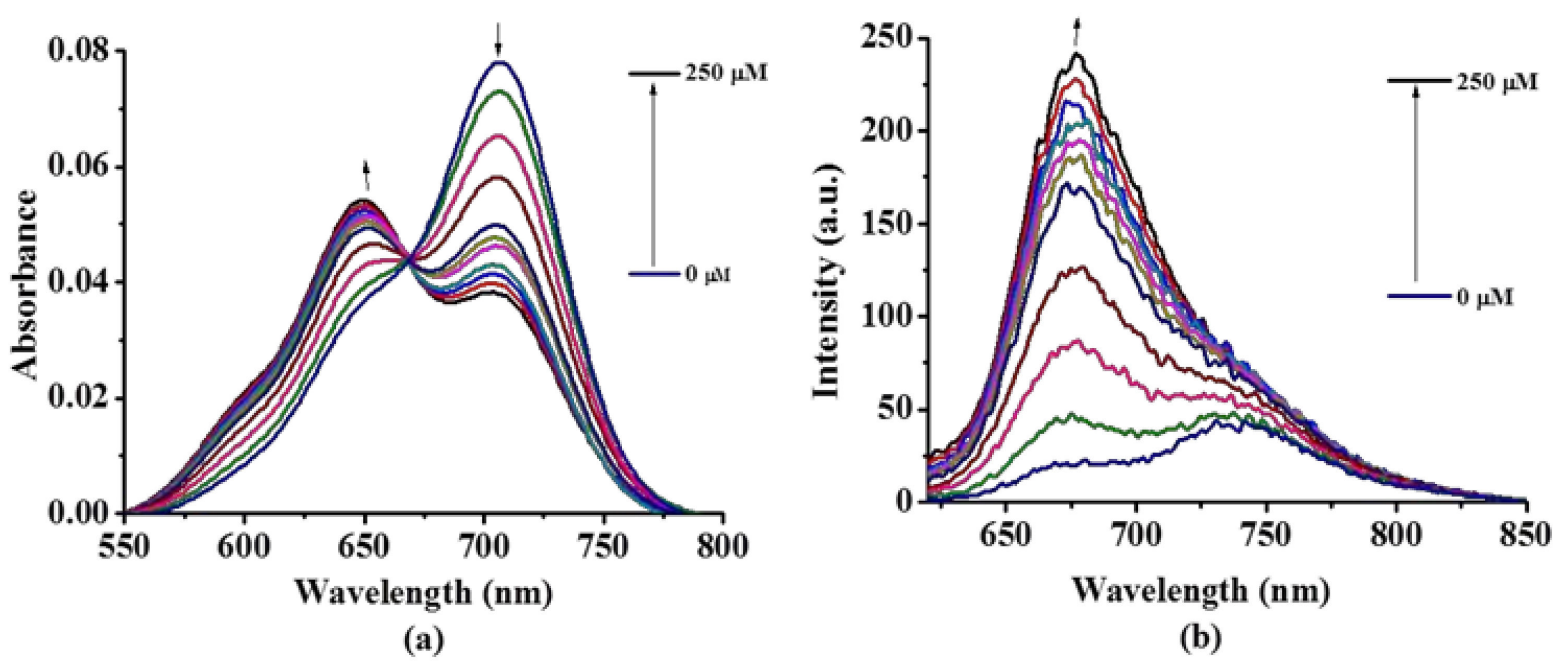
(**a**) Absorption and (**b**) fluorescence emission spectra of compound **7** (1 μM) with different concentrations of H2O2 in phosphate buffer solution (10 mM, pH 7.4) with 0.05% Tween 80.

**Figure 4:**
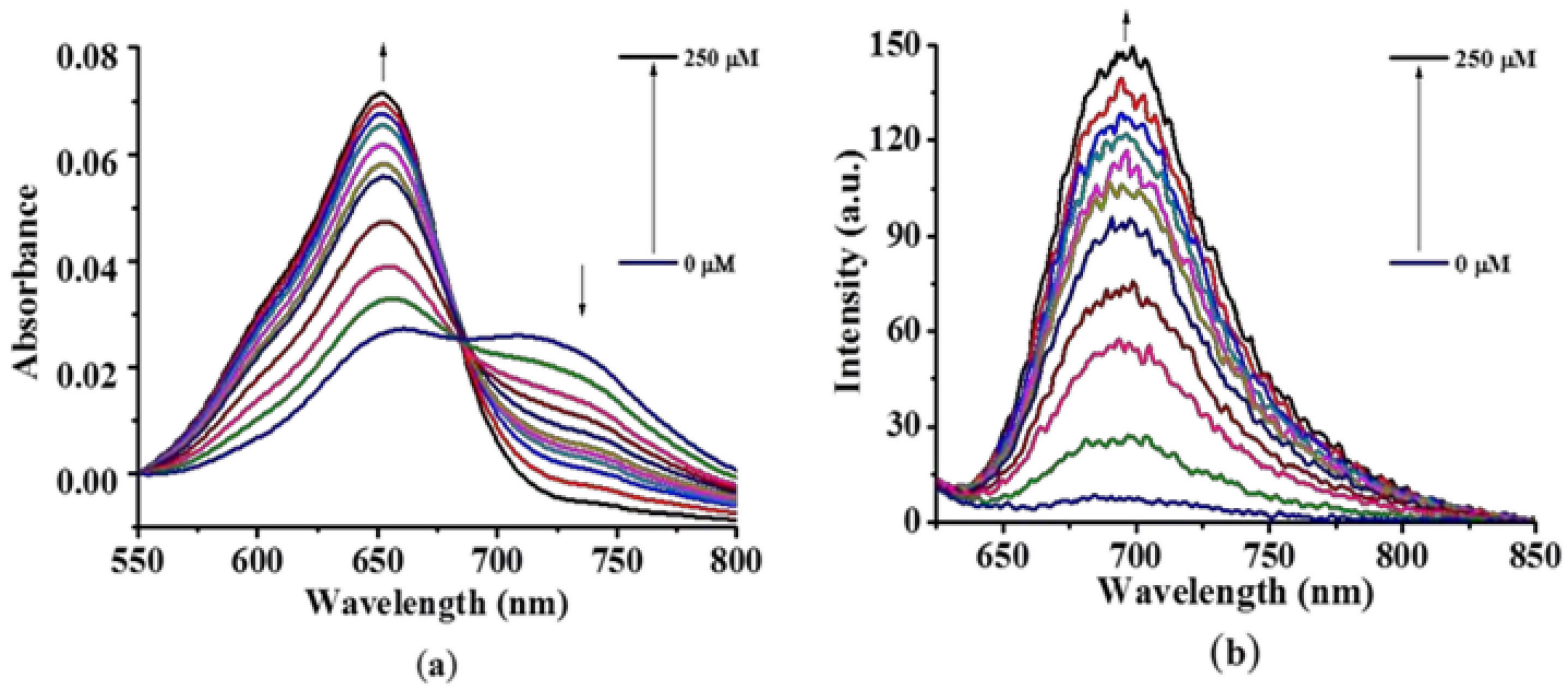
(**a**) Absorption and (**b**) fluorescence emission spectra of compound **7** (1 μM) with different concentration of H_2_O_2_ in solution (PBS/DMSO = 4:1, *v*/*v*; 10 mM, pH = 7.4).

**Figure 5:**
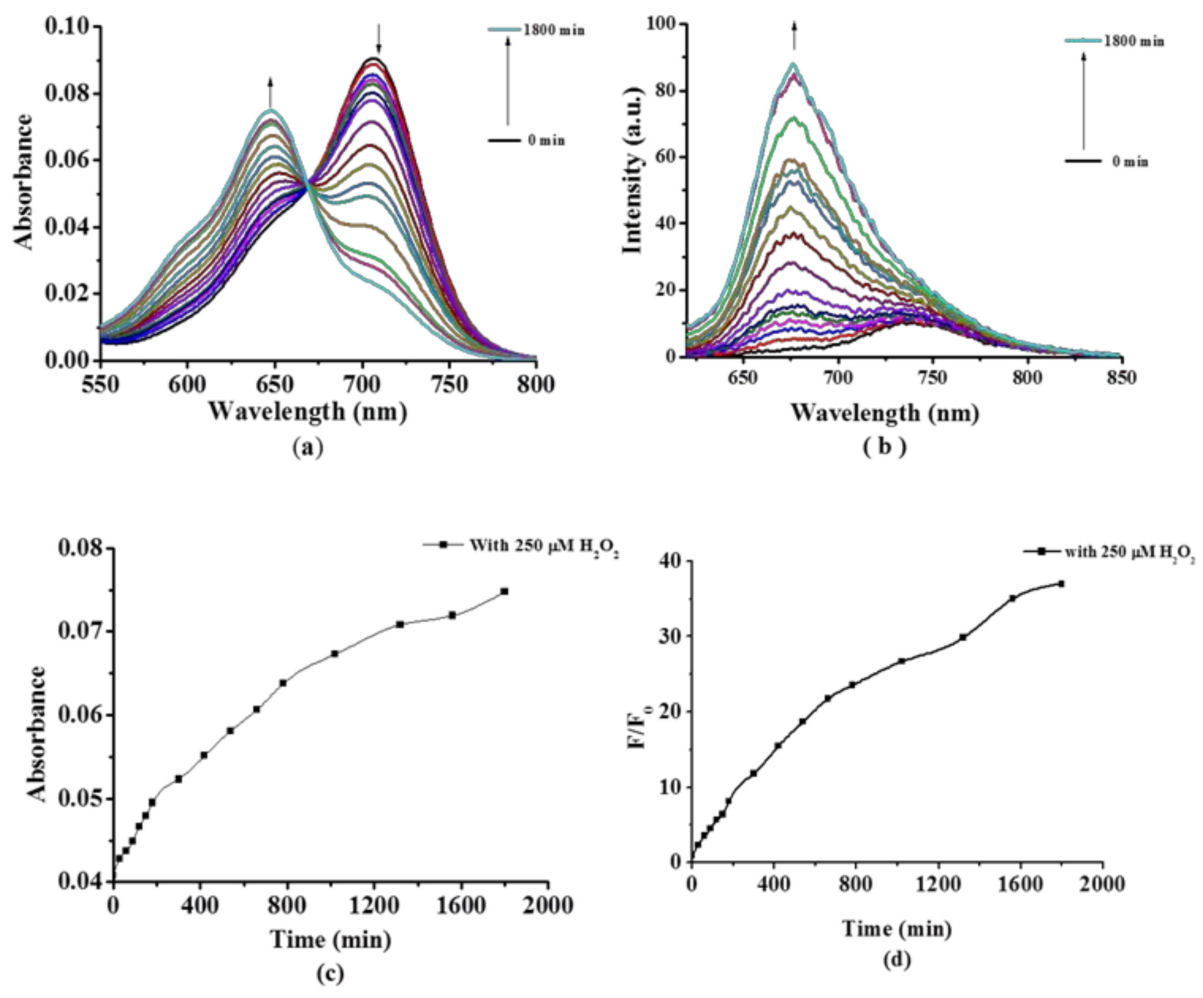
(**a**) Absorption and (**b**) fluorescence emission spectra for compound **7** (1 μM) upon incubating with H_2_O_2_ (250 μM) for varying time intervals; the plots show the time-dependent increases (**c**) absorption at 647 nm and (**d**) fluorescence intensities at 678 nm; the experiments are carried out in phosphate buffer solution (10 mM, pH 7.4) containing 0.05% Tween 8.

**Figure 6:**
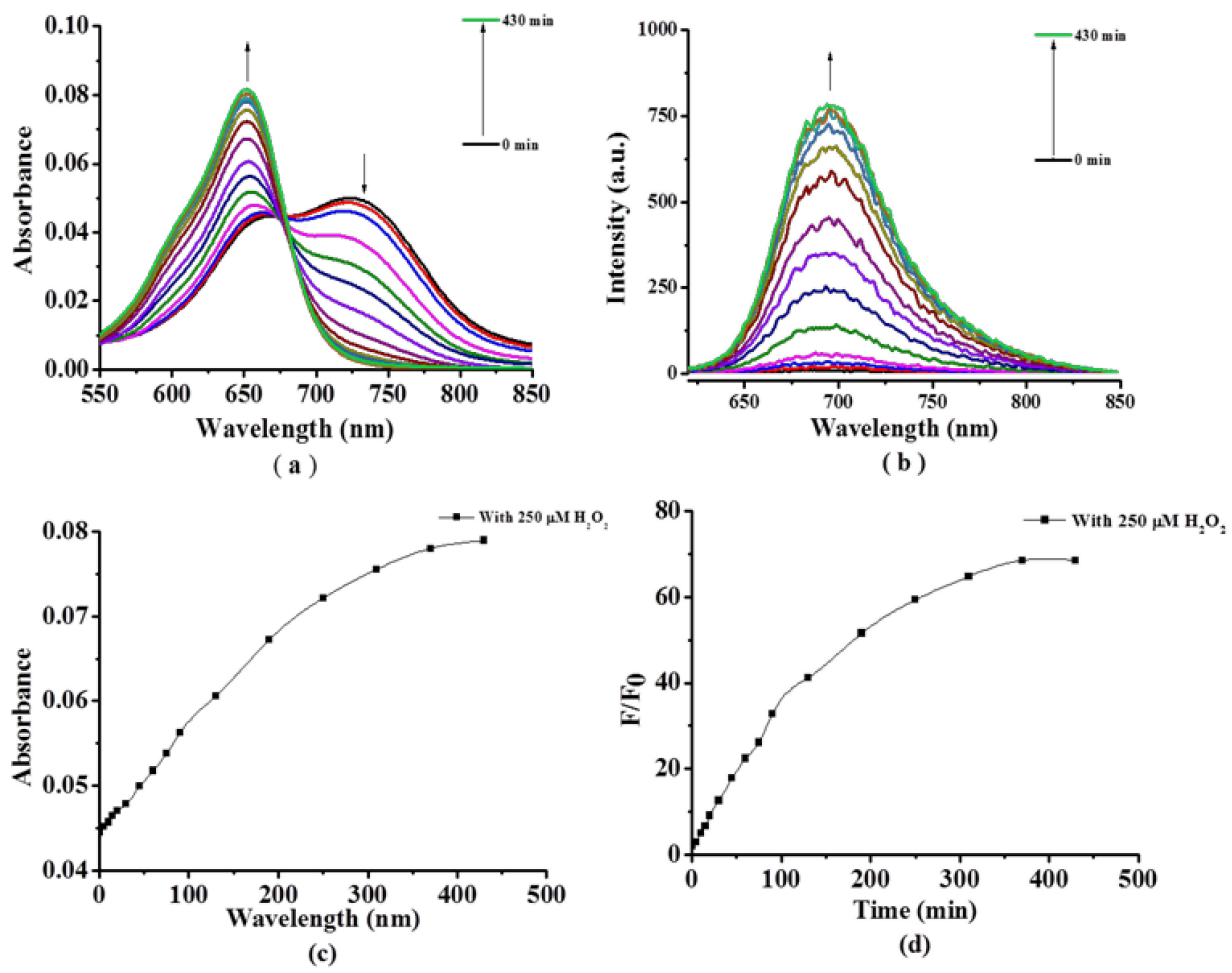
(**a**) Absorption and (**b**) fluorescence emission spectra for compound **7** (1 μM) upon incubating with H_2_O_2_ (250 μM) for varying time intervals; the plots show the time-dependent increases (**c**) absorption at 653 nm and (**d**) fluorescence intensities at 678 nm; the experiments are carried out in solution (PBS/DMSO = 4:1, *v*/*v*; 10 mM, pH = 7.4).

For the purpose of studying the effects of H_2_O_2_ on the spectral properties of compound **7**, we performed a series of tests with different H_2_O_2_ concentrations in PBS solution (Figure 3) and PBS buffer solution containing 20% DMSO (Figure 4), respectively. As shown in Figure 3a, there is significant blue-shift in the absorption spectra of **7** upon addition of H_2_O_2_ (incubated for 8 h). The blue spectral shifts is due to “ester-to-carboxylate” conversion, as depicted in Scheme 1. As a result of conversion, the intramolecular PET is inhibited, the fluorescence of BODIPY dye is recovered (Figure 3b). The same phenomenon is observed in Figure 4. The effect of incubation time on the absorption and fluorescence spectra of compound **7** has also been explored (the concentration of H_2_O_2_ is fixed at 250 μM). As shown in Figure 5 and Figure 6, an obvious blue-shifted absorption and enhanced fluorescence emission are observed. The fluorescence intensity of compound **7** at 678 nm increases more than 40 times after interacted with 250 μM H_2_O_2_ in a mixture of PBS and DMSO (4:1, *v*/*v*) for several hours. All the results suggest that the arylboronate bond of compound **7** is susceptible to hydrogen peroxide mediated cleavage and releases *meso*-carboxylate-substituted BODIPY that has strong fluorescence, which depends on the H_2_O_2_ concentrations and incubation time.

**Figure 7:**
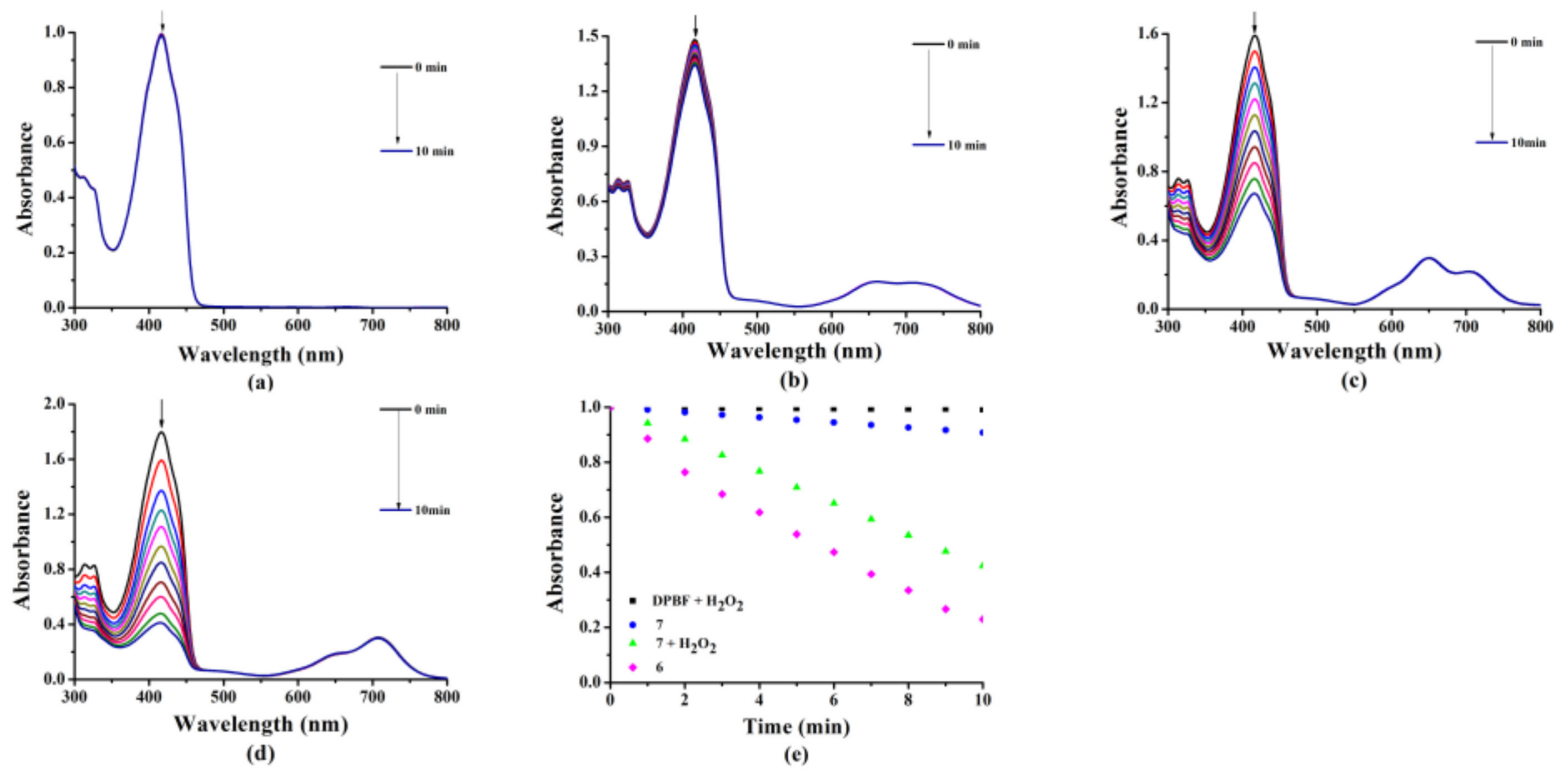
The absorption spectra of DPBF (0.1 μM) after different durations of 670 nm laser irradiation (**a**) only DPBF in the presence of H2O2; (**b**) with compound **7** (5 μM); (**c**) with compound **7** (5 μM) in the presence of H2O2 (250 μM); (**d**) compound **6** (5 μM); (**e**) comparison of the degradation rates of 1,3-diphenylisobenzofuran (DPBF) as monitored by the decrease in absorbance at 415 nm with irradiation time; the experiments are carried out in solution (PBS/DMSO = 4:1, *v*/*v*; 10 mM, pH = 7.4).

**Figure 8:**
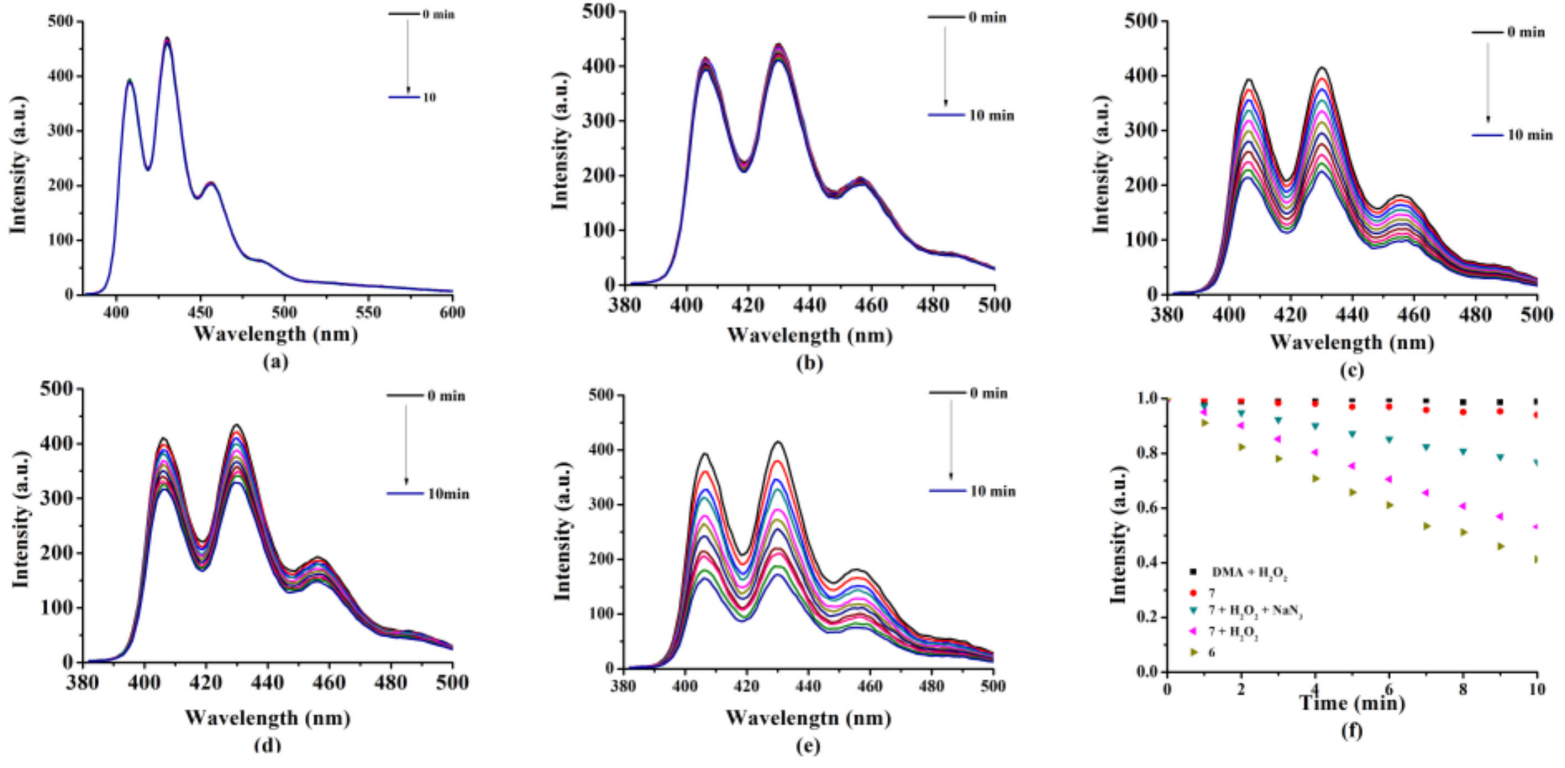
The fluorescence emission spectra of DMA (6 μM) after different durations of 670 nm laser irradiation (**a**) only DMA in the presence of H_2_O_2_; (**b**) with compound **7** (5 μM); (**c**) with compound **7** (5 μM) in the presence of H_2_O_2_ (250 μM); (**d**) with compound **7** (5 μM) in the presence of H_2_O_2_ (250 μM) and NaN_3_; (**e**) with compound **6** (5 μM) in the absence of H_2_O_2_.; (**f**) comparison of the degradation rates of DMA as monitored by the decrease in fluorescence intensity at 432 nm (excited at 370 nm) with irradiation time; the experiments are carried out in phosphate buffer solution (10 mM, pH 7.4) containing 0.05% Tween 80.

We adopt the 1,3-diphenylisobenzofuran (DPBF) as an indicator to study the effect of H_2_O_2_ on ^1^O_2_ generation efficiencies of compound **7** in a mixture of DMSO and PBS (1:4, *v*/*v*) by monitoring the absorption changes of DPBF at 415 nm. When only treated with compound **7**, followed by light irradiation, the absorbance of DPBF at 415 nm has a negligible change (Figure 7b,e). However, treated compound **7** in the presence of H_2_O_2_, the absorbance of DPBF at 415 nm decreases gradually (Figure 7c,e). This is because that compound **7** is activated by H_2_O_2_ to generate ^1^O_2_ inducing degradation of DPBF. By contrast, compound **7** treated with H_2_O_2_ (250 μM) exhibited a slight lower ^1^O_2_ generation efficiency than *meso*-carboxylate-substituted BODIPY **6** (Figure 7c–e). The same experimental results are obtained in PBS with 9,10-dimerthylanthracence (DMA) as the singlet oxygen indicator (Figure 8a–e). Pretreated compound **7** with H_2_O_2_, upon illumination, the fluorescence of DMA at 432 nm decreases gradually (Figure 8b,e). However, in the absence of H_2_O_2_, the fluorescence of DMA at 432 nm exhibits a negligible change (Figure 8a,e). Besides, we use NaN_3_ as a scavenger for ^1^O_2_ to get that the degradation rate of 9,10-dimerthylanthracence (DMA) is slowed down (Figure 8c,e). It is believed that the arylboronate group of the compound **7** quenches most of their singlet excited state, reducing the fluorescence quantum yield and singlet oxygen generation efficiency, and the H_2_O_2_ can activate them.

### 2.3. In Vitro Photodynamic Activity Study

#### 2.3.1. Cellular Fluorescence Imaging

**Figure 9:**
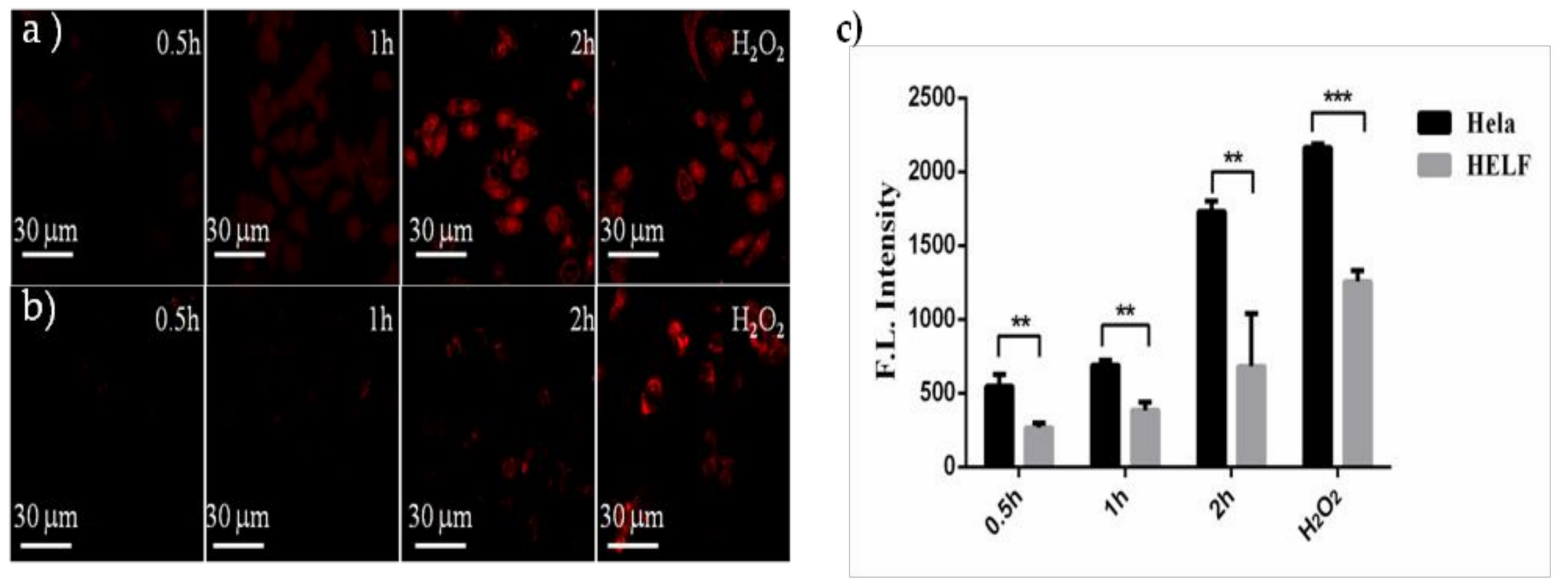
CLSM images of (**a**) HeLa cells and (**b**) HELF cells after incubation with compound **7** (5 μM) for 0.5 h, 1 h, and 2 h respectively, or cells after incubation with H_2_O_2_ (50 μM) for 0.5 h, followed by treatment of the compound **7** (5 μM) for another 0.5 h; (**c**) quantitative results of (**a**) and (**b**) (** *p* < 0.01, *** *p* < 0.001).

To study the activation of compound **7** by H_2_O_2_ at cellular level, we use the confocal laser scanning microscopy (CLSM) to observe the intracellular fluorescence imaging by incubating HeLa and HELF cells with compound **7** (5 μM) for different time. As illustrated in Figure 9a, after treatment with compound **7** in HeLa cancer cells, the intracellular fluorescence intensity increases gradually with the incubation time. This indicates that the intracellular uptake of compound **7** is time-dependent and compound **7** is efficiently activated by endogenous H_2_O_2_ in the cancer cells. By contrast, the fluorescence intensity in HELF normal cells is much lower than that in HeLa cancer cells (Figure 9a–c), namely, the amount of intracellular H_2_O_2_ determines the extent and rate at which the arylboronate group of compound **7** is cleaved. Those results suggesting the compound **7** is a promising photodynamic agent with good ability to target cancer cells. In addition, we incubated those two kinds of cells with exogenous H_2_O_2_ (50 μM) for 0.5 h initially, followed by compound **7** (5 μM) for another 0.5 h. Obviously, the intracellular fluorescence intensity significantly increases in both two types of cells.

#### 2.3.2. Measurements of Intracellular ROS

**Figure 10:**
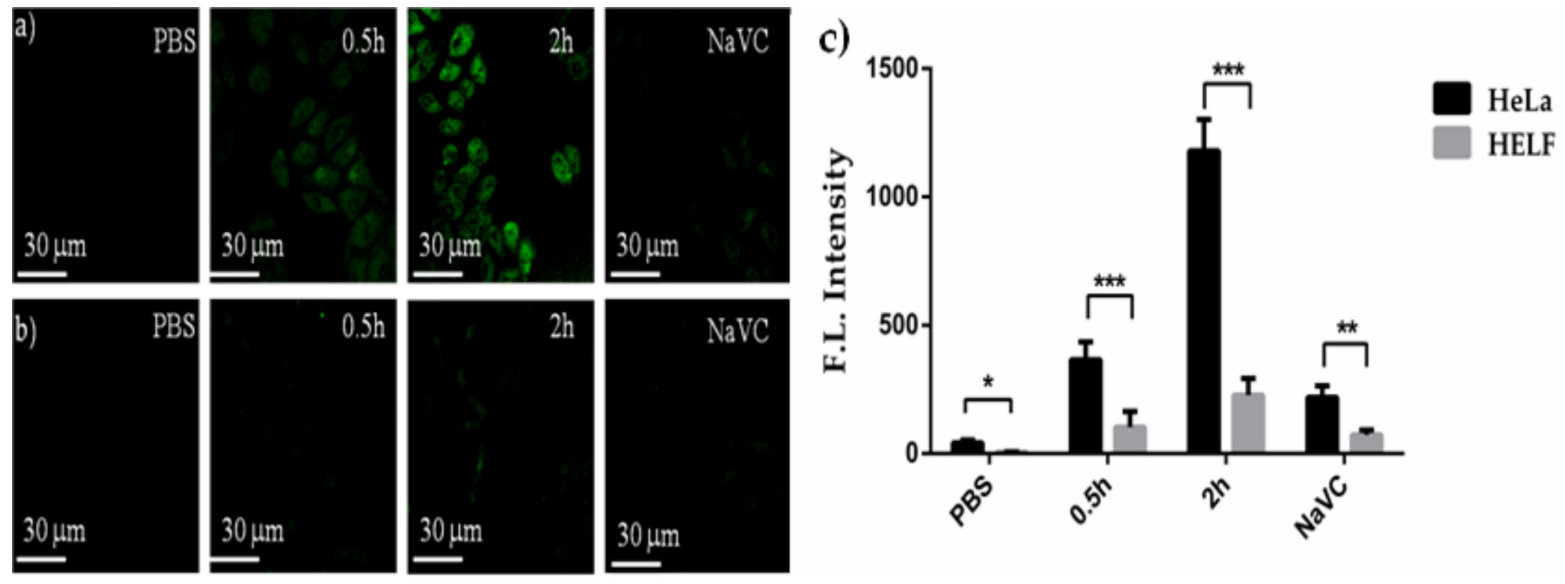
CLSM images of (**a**) HeLa cells and (**b**) HELF cells incubated with compound **7** (5 μM) for 0.5 h and 2 h respectively, or cells by co-culturing with compound **7** (5 μM) and NaVC (100 μM) for 2 h and the PBS group used as a control to evaluate the basal ROS levels in the absence of compound **7**. The green fluorescence is DCF signals; (**c**) quantitative results of (**a**) and (**b**) (* *p* < 0.05, ** *p* < 0.01, *** *p* < 0.001).

Here, the 2′,7′-dichlorofluorescein diacetate (DCFH-DA) was employed as an indicator to detect the generation of intracellular ROS. It is well known that DCFH-DA itself is nonfluorecent, but it can be oxidized by ROS into 2′,7′-dichlorofluorescein (DCF) with intense green fluorescence [40]. Both HeLa and HELF cells were firstly treated with compound **7** for 0.5 or 2 h, and then illuminated by red light using PBS as a control. As shown in Figure 10, there is a significantly weak fluorescence in HELF cells, whereas a remarkable green fluorescence is observed in HeLa cancer cells, indicating the prescence of high concentrations of ROS in HeLa cells. In addition, the fluorescence intensity increases with incubation time. However, after treated with compound **7** (5 μM) and sodium vitamin C (NaVC) as a ROS scavenger (100 μM) for 2 h, the CLSM images of the HeLa only show a weak green fluorescence. The above results indicate that compound **7** can be activated and have the high ROS generation ability in HeLa cells.

#### 2.3.3. Cytotoxicity Studies

**Figure 11:**
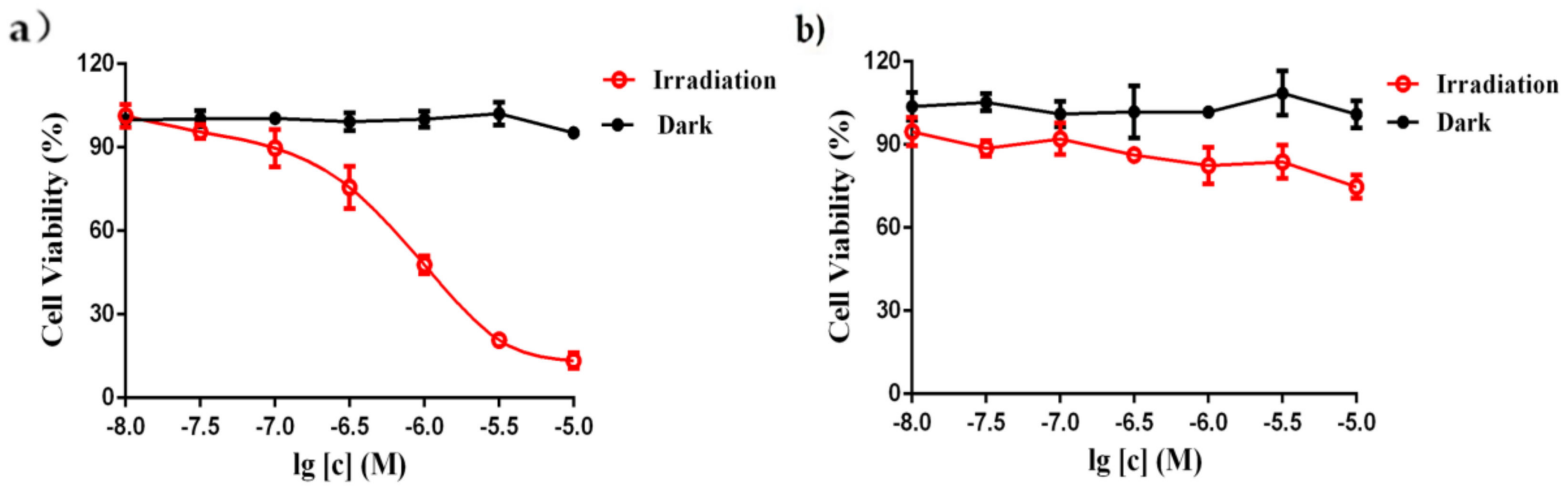
Cytotoxic effects of compound **7** on (**a**) HeLa and (**b**) HELF cells in the absence (black) and presence (red) of light (λ = 670 nm, 20 mW∙cm^−2^, 2.4 J∙cm^−2^). Data are expressed as the mean standard error of the means of three independent experiments, each performed in sextuplicate.

The cytotoxicity of compound **7** was evaluated by MTT assay using HeLa and HELF cells. As shown in Figure 11b, compound **7** has almost negligible toxicity against HELF cells in the presence of or in absence of light, that is, there is neither phototoxicity nor dark toxicity to HELF cells for compound **7**. This is because that compound **7** has not been activated in the normal cells with a relatively low concentration of H_2_O_2_, which leads to significantly low phototoxicity. Also, compound **7** hardly damaged HeLa cells in absence of light (Figure 11b). However, when there is light, the cell viability of HeLa cells is significantly reduced as a result of photodynamic activity of compound **7** having been restored in HeLa cells (Figure 11b). The corresponding IC_50_ value, defined as the dye concentration required killing 50% of the cells, is 0.95 μM. All above demonstrate that the photodynamic activity of compound **7** is related to the concentration of H_2_O_2_, and has good phototoxicity against cancer cells with high H_2_O_2_ concentration. Compound **7** does no damage to normal cells even in the presence of light, indicating that the photosensitizer we designed has good specificity for cancer cells against normal cells.

#### 2.3.4. Subcellular Localization Studies

**Figure 12:**
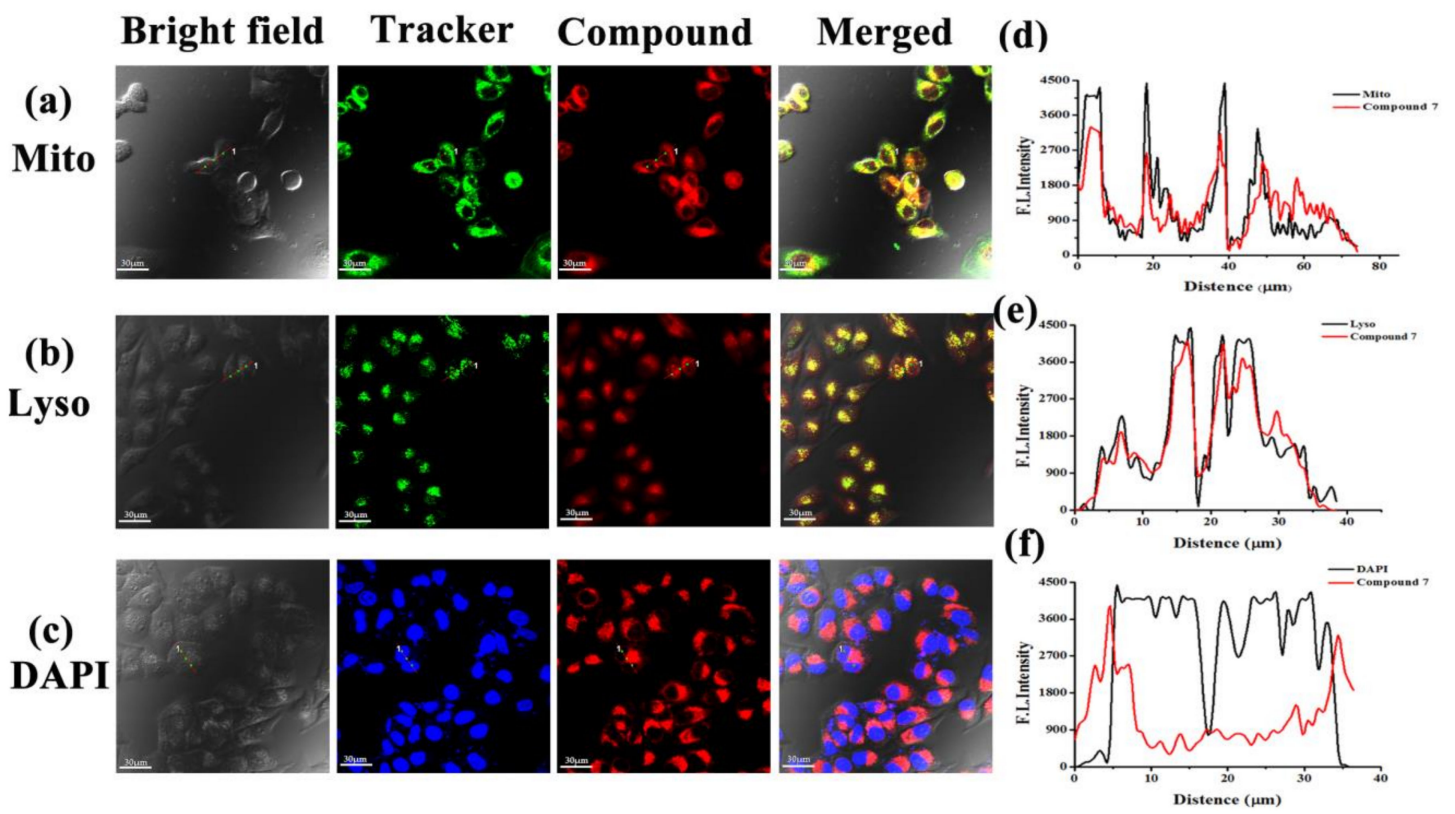
Visualization of the intracellular fluorescence of HeLa cells for Mito-Tracker Green (in green, **a**), Lyso-Tracker (in green, **b**), DAPI (in blue, **c**), and compound **7** (in red, 5 μM). Fluorescence intensity profiles of DAPI (**d**), Mito-Tracker Green (**e**), Lyso-Tracker (**f**), and compound **7** (5 μM) traced along the red line in (**a**, **b**, **c**).

The subcellular localization of compound **7** in HeLa cells was also studied. From the previous literatures, we get that nucleus, mitochondria and lysosomes play important roles in photodynamic therapy. Therefore, we stained the cells with compound **7** together with DAPI, Mito-Tracker Green and Lyso-Tracker which are specific fluorescence probes for nucleus, mitochondria and lysosomes, respectively. As depicted in Figure 12a, the fluorescence of Mito-Tracker Green and compound **7** are well superimposed. And the line traces in the Figure 11d also illustrate this conclusion. In addition, the experimental results in lysosomes are similar to those of mitochondria (Figure 12b,e). By contrast, the fluorescence images of compound **7** and the DAPI cannot be superimposed (Figure 12c,f). As the previous studies reported [41], intracellular hydrogen peroxide is mainly produced in the mitochondria and then spread into lysosomes. And so, H_2_O_2_-mediated cleavage of compound **7** can occur in mitochondria and lysosomes.

## 3. Materials and Methods

### 3.1. General Information

Most of the chemical reagents and solvents were purchased from Energy Chemical Technology Co., Ltd. (Shanghai, China) except for otherwise stated. Chromatographic purifications were performed on silica gel (Qingdao Ocean, Qingdao, Shandong, China, 200–300 mesh) columns with the indicated eluents. The two kinds of cells, human cervical carcinoma (HeLa) and human embryonic lung fibroblast (HELF) cells were obtained from the cell bank of Shanghai Institutes for Biologic Sciences. And 3-(4,5-dimethylthiazol-2-yl)-2,5-diphenyltetrazolium bromide (MTT) was purchased from Gen View Co., Ltd., Tallahassee, FL, USA. Compounds **1, 2**, **3** [37] were prepared as described.

NMR spectra were recorded on a Bruker Avance III 400 (^1^H: 400 MHz, ^13^C: 100.6 MHz) instrument (Bruker, Karlstuhe, Germany) and referenced to tetramethylsilane (TMS) as the internal standard. High-resolution mass spectra (HRMS) analysis was carried out on an Agilent 6520 ACURATE-Mass Q-TOF Mass Spectra (Agilent Technologies, Santa Clara, CA, USA). Electronic absorption spectra were measured on a Lambda 365 UV-Visible absorption spectrometer (Perking Elmer, Waltham, MA, USA) and fluorescence spectra were obtained on a VARIAN Carye Elipse Fluorescence spectrometer (Agilent Technologies, Santa Clara, CA, USA). Intracellular fluorescence imaging and subcellular localization was carried on an Olympus FV1000 Confocal Laser Scanning Microscope (Olympus Instrument Co., Ltd., Shinjuku-ku, and Tokyo, Japan).

### 3.2. Chemistry

#### 3.2.1. Synthesis of Compound **4**

Compound **3** (0.060 g, 0.20 mmol) was dissolved in absolute ethanol (200 mL), and then added the iodine (0.17 g, 0.67 mmol) and iodic acid (0.10 g, 0.55 mmol). After magnetic stirring under an atmosphere of nitrogen at 60 °С for 4 h, ethanol was removed under reduced pressure. The residue was chromatographed on silica gel with petroleum ether and dichloromethane (1/1, *v*/*v*) as eluent to give the red solid compound **4**. ^1^H-NMR (400 MHz, CDCl_3_): δ (ppm) 4.00 (s, 3H, OCH_3_), 2.62 (s, 6H, CH_3_), 2.13 (s, 6H, CH_3_). ^13^C-NMR (100.6 MHz, CDCl_3_): δ (ppm) 165.2, 158.8, 143.5, 128.4, 128.2, 85.6, 53.4, 16.2, 15.2; HRMS (ESI): *m*/*z* calcd. for C_15_H_15_BF_2_I_2_N_2_NaO_2_ [M + Na]^+^, 580.9176; found, 580.9238 (See supplementary materials).

#### 3.2.2. Synthesis of Compound **5**

Compound **4** (0.20 g, 0.36 mmol) was dissolved in a two-neck round bottom flask with 100 mL toluene, and followed by the compound **2** (0.65 g, 1.52 mmol), piperidine (1.2 mL), glacial acetic acid (1.0 mL) and an appropriate amount of anhydrous magnesium perchlorate. Under nitrogen protection, it was condensed and refluxed for 2.5 h with a water separator. After that, the toluene was evaporated under reduced pressure. The residue was mixed with water, and extracted three times by dichloromethane. The combined organic fractions were collected and dried under reduced pressure. The residue was chromatographed on silica gel with dichloromethane and methanol (50:1, *v*/*v*) as eluent to afford green viscous compound **5** (0.19 g, 39%). ^1^H-NMR (400 MHz, CDCl_3_): δ (ppm) 8.14 (d, *J* = 16.8 Hz, 2H, CH=CH), 7.51 (d, *J =* 16.8 Hz, 2H, CH=CH), 7.30 (d, *J =* 8.4 Hz, 2H, ArH), 7.18 (s, 2H, ArH), 6.97 (d, *J =* 8.4 Hz, 2H, ArH), 4.30–4.22 (m, 8H, OCH_2_), 4.03 (s, 3H, OCH_3_), 3.95–3.88 (m, 8H, OCH_2_), 3.81–3.75 (m, 8H, OCH_2_), 3.73–3.64 (m, 16H, OCH_2_), 3.60–3.52 (m, 8H, OCH_2_), 3.40 (s, 6H, OCH_3_), 3.38 (s, 6H, OCH_3_), 2.23 (s, 6H, CH_3_). ^13^C-NMR (100.6 MHz, CDCl_3_): δ (ppm) 165.8, 151.7, 150.7, 148.9, 143.5, 140.3, 130.4, 130.2, 125.1, 122.0, 116.9, 114.2, 114.1, 83.0, 71.91, 71.89, 70.83, 70.81, 70.6, 70.51, 70.48, 69.7, 69.6, 69.1, 68.7, 59.00, 58.97, 53.4, 15.5; HRMS (ESI): *m*/*z* calcd. for C_57_H_80_BF_2_I_2_N_2_O_18_ [M + H]^+^, 1383.3551; found, 1383.3673.

#### 3.2.3. Synthesis of Compound **6**

To a solution of compound **5** (0.26 g, 0.19 mmol) in ethyl acetate (100 mL) was added lithium iodide (0.13 g, 0.95 mmol). The resulting mixture was refluxed overnight under an atmosphere of nitrogen. Then the mixture was washed three times with water. The solvent was removed under reduced pressure. The residue was further purified by a silica gel column chromatography using dichloromethane and methanol (25:1, *v*/*v*) as eluent to give green viscous compound **6** (0.20 g, 78%). ^1^H-NMR (400 MHz, CDCl_3_): δ (ppm) 7.98 (d, *J =* 16.8 Hz, 2H, CH=CH), 7.45 (d, *J =* 16.8 Hz, 2H, CH=CH), 7.20 (d, *J =* 7.6 Hz, 2H, ArH), 7.06 (s, 2H, ArH), 6.86 (d, *J =* 7.6 Hz, 2H, ArH), 4.22–4.06 (m, 8H, OCH_2_), 3.89–3.76 (m, 8H, OCH_2_), 3.72–3.57 (m, 24H, OCH_2_), 3.53–3.46 (m, 8H, OCH_2_), 3.32 (s, 6H, OCH_3_), 3.30 (s, 6H, OCH_3_), 2.45 (s, 6H, CH_3_). ^13^C-NMR (100.6 MHz, MeOD): δ (ppm) 168.7, 149.9, 149.5, 148.4, 144.3, 138.2, 130.2, 129.5, 121.7, 117.0, 113.6, 112.9, 81.0, 71.39, 71.37, 70.1, 70.0, 69.82, 69.76, 69.7, 69.1, 68.9, 68.5, 68.2, 57.87, 57.85, 14.9; HRMS(ESI): *m*/*z* calcd. for C_56_H_77_BF_2_I_2_N_2_NaO_18_ [M + Na]^+^, 1391.3214; found, 1391.3339.

#### 3.2.4. Synthesis of Compound **7**

Compound **6** (0.048 g, 0.035 mmol) was dissolved in 10 mL of anhydrous tetrahydrofuran in a round flask, followed by 4-bromomethylphenylboronic acid pinacol ester (0.021 g, 0.073 mmol), 1,8-diaza Heterobicyclo[5.4.0] undec-7-ene (0.017 g, 0.11 mmol) and an appropriate amount of potassium iodide. The mixture was stirring overnight under nitrogen. Then the mixture was mixed with water and then extracted three times with dichloromethane. The combined organic fractions were collected and dried under reduced pressure. The residue was further purified by a silica gel column chromatography using dichloromethane and methanol (50:1, *v*/*v*) as eluent to afford green viscous compound **7** (0.022 g, 39%). ^1^H-NMR (400 MHz, CDCl_3_): δ (ppm) 8.10 (d, *J =* 16.8 Hz, 2H, CH=CH), 7.85 (d, *J =* 8.4 Hz, 2H, ArH), 7.47 (d, *J =* 16.8 Hz, 2H, CH=CH), 7.44 (d, *J =* 8.4 Hz, 2H, ArH), 7.29–7.24 (m, 2H, ArH), 7.15 (s, 2H, ArH), 6.94 (d, *J =* 8.4 Hz, 2H, ArH), 5.43 (s, 2H, OCH_2_), 4.27–4.19 (m, 8H, OCH_2_), 3.92–3.86 (m, 8H, OCH_2_), 3.79–3.72 (m, 8H, OCH_2_), 3.70–3.60 (m, 16H, OCH_2_), 3.56–3.50 (m, 8H, OCH_2_), 3.37 (s, 6H, OCH_3_), 3.45 (s, 6H, OCH_3_), 2.12 (s, 6H, CH_3_), 1.36 (s, 12H, CH_3_). ^13^C-NMR (100.6 MHz, CDCl_3_): δ (ppm) 165.1, 151.7, 150.7, 148.9, 143.6, 140.2, 136.1, 135.3, 134.8, 130.4, 130.2, 129.9, 128.4, 128.2, 122.0, 117.0, 114.2, 84.0, 83.0, 71.9, 70.8, 70.6, 70.49, 70.46, 69.7, 69.6, 69.1, 68.7, 58.98, 58.95, 24.9, 15.7; HRMS(ESI): *m*/*z* calcd. for C_69_H_94_B_2_F_2_I_2_N_2_NaO_20_ [M + Na]^+^, 1607.4536; found, 1607.4780.

### 3.3. Photo-Physical and Photo-Chemical Studies

#### 3.3.1. Absorption and Fluorescence Studies

The electronic absorption spectra were measured on a Lambda 365 UV-Visible absorption spectrometer (Perking Elmer, Shanghai, China) and fluorescence spectra were obtained on a VARIAN Carye Elipse Fluorescence spectrometer (Agilent Technologies, Santa Clara, CA, USA) by using the compounds dissolving in DMSO as described. And we calculated the fluorescence quantum yield using the following formula:

(1) $${Ф}_{F}={Ф}_{F}^{\mathrm{std}\;}\times \;\frac{F}{F_{\mathrm{std}}}\times \;\frac{A_{\mathrm{std}}}{A}\times \left(\frac{ŋ}{{ŋ}_{\mathrm{std}}}\right)²$$

where F and F_std_ are the measured fluorescence area (λ_ex_ = 610 nm) of the compounds and standard; A and A_std_ are the absorbances at the excitation wavelength (610 nm) of the compounds and standard; η and η_std_ are the refractive index of the compounds and standard. Here, the unsubstituted zinc (II) phthalocyanine (ZnPc) in DMF was used as the standard [${Ф}_{F}^{\mathrm{std}}$ = 0.28]. To minimize re-absorption of the radiation by the ground-state species, the emission spectra were obtained by controlling the absorbances of compounds and ZnPc at 610 nm were within the range from 0.03 to 0.05.

#### 3.3.2. Detection of Singlet Oxygen (^1^O_2_) Generation Efficiency

Singlet oxygen generation efficiency was indirectly measured by using the 1,3-diphenylisobenzofuran (DPBF) in PBS:DMSO (4:1, *v*/*v*) and 9,10-dimethylanthracen (DMA) in phosphate buffer solution (10 mM, pH 7.4) containing 0.05% Tween 80 as the scavengers, respectively. After incubation for 4 h, the mixture of DPBF (1 μM), photosensitizer (5 μM) and different concentrations of H_2_O_2_ in DMSO:PBS (1:4, *v*/*v*) was illuminated with laser (670 nm, 80 mW·cm^−2^). The decay of the DPBF absorption at 415 nm was monitored. Similarly, the mixture of DMA (6 μM), photosensitizer (5 μM) and different concentrations of H_2_O_2_ in PBS was illuminated with laser (670 nm, 80 mW·cm^−2^). The decay of the DMA fluorescence intensity at 432 nm was monitored (λ_ex_=370 nm).

### 3.4. In Vitro Studies

#### 3.4.1. Cell Culture and Conditions

Human embryonic lung fibroblast (HELF) and human cervical carcinoma (HeLa) cells were maintained in Dulbecco’s modified Eagle’s medium (DMEM) (Gibco) supplemented with Fetal Calf Serum (10%, *v*/*v*), penicillin streptomycin (1%, *v*/*v*) at 37 °С in a humidified atmosphere with 5% CO_2_. Approximately 7 × 10^3^ (for HeLa) cells per well in these media were inoculated in 96-multiwell plates and incubated overnight at 37 °С in a humidified 5% CO_2_ atmosphere. Then, we collected selectively those cells for following use.

#### 3.4.2. Photocytotoxicity Studies

Compound **7** was first dissolved in DMSO to obtain 1 mM solutions, which then were diluted with the culture medium to appropriate concentrations (0, 0.01, 0.05, 0.1, 0.5, 1, 3, 6, 9 μM) Wherein the DMSO content in the final diluted drug solution does not exceed 1% (*v*/*v*). The cells were incubated with medium containing 1% DMSO in either the light or non-lighted cell control group to deduct the toxic effects of DMSO on the cells. And then select suitable HeLa and HELF cells and seeded them in 96-well plates. After incubation for 24 h, different concentrations of compound **7** were added, followed by incubating with compound **7** for 24 h, and rinsing twice with PBS, and finally fresh medium was added. The fluence rate of the light source (λ = 670 nm) was 20 mW·cm^−2^.

The toxicity of compound **7** against HeLa and HELF cells was determined by MTT assay. After illumination for 2 min, the experimental group continued to incubate for 24 h. And the control group, after being rinsed twice with PBS, were incubated for 24 h. After that, a prepared 10 μL MTT (5 mg/mL) solution was added to each well. The cells then were incubated under 5% CO_2_ at 37 °C for 4 h. The solution in the 96-well plate was removed, and then 100 μL/well of DMSO was added. In addition, 100 μL of DMSO was also added as a blank group in the wells without cells, and the light at λ = 490 nm was read with a microplate reader.

According to the previous work [42], the cell viability was then determined by the following equation: % viability=[∑(A_i_/A_control_ × 100)]/n, where Ai is the absorbance of the ith data (i = 1, 2,..., n), A_control_ is the average absorbance of the control wells in which the BODIPY was absent, and n (= 6) is the number of the data points. Six replicates were used for each concentration in each experiment and each experiment was repeated three times. The procedures for investigation of dark-cytotoxicity were almost the same except that there was no irradiation. The dose-dependent survival curves were prepared with GraphPad Prism 5.0 Software (GraphPad Software Inc., La Jalla, CA, USA) and IC_50_ was calculated.

#### 3.4.3. Measurements of Intracellular ROS

The detection of ROS is based on the indirect verification by DCFH-DA, which is non-fluorescent while can be oxidized to form the fluorescent compound DCF.

First of all, HELF and HeLa cells were seed in a CLSM-special cell dishes at 1.0 × 10^5^ cells per dish. After incubation for 24 h, compound **7** (5 μM was added, and the cells in different groups were ingested for compound **7** (λ_ex_ = 635 nm, λ_em_ = 640–800 nm) for 0.5 h, 1 h, 2 h, respectively. Afterwards, DCFH-DA (λ_ex_ = 488 nm, λ_em_ = 500–550 nm), a probe for capturing ROS, was added and co-cultured with the cells. After half an hour, the cells were rinsed with PBS for 5 times, and irradiated for 2 min; then confocal fluorescence imaging was performed to give the level of intracellular ROS with the excitation wavelength of 488 nm and emission wavelength from 500 to 550 nm.

#### 3.4.4. Subcellular Localization Studies

HeLa cells in good condition were seed in a CLSM-special cell dishes at 1.0 × 10^3^ cells per dish. After incubation for 24 h, compound **7** (5 μM) was added and incubated for another 24 h. After being washed with PBS, the cells were incubated with 2 mL of DAPI, Mito-Tracker, or Lyso-Tracker in culture medium for 30 min. Then the cells were rinsed with PBS five times again and viewed with an Olympus FV1000 Confocal Laser Scanning Microscope (Olympus Instrument Co., Ltd., Shinjuku-ku, Tokyo, Japan). And the images were collected with the excitation wavelength of 405 nm (DAPI), 488 nm (Mito-Tracker Green), 488 nm (Lyso-Tracker DNd-26) and 633 nm (compound **7**), and the fluorescence emission wavelength of 425–475 nm (blue, DAPI), 510–570 nm (green, Mito-Tracker Green), 510–570 nm (green, Lyso-Tracker DNd-26), and 650–750 nm (red, compound **7**).

## 4. Conclusions

In conclusion, we have prepared a novel H_2_O_2_-responsive BODIPY-based photosensitizer which is caged by an arylboronate group via PET mechanism. This arylboronate-linked BODIPY shows enhanced fluorescence emission and ROS generation in the presence of H_2_O_2_, both in PBS and inside tumor cells, due to cleavage of the arylboronate group linker. With these advantages, this compound is worthy of further study as a potential theranostic agent for cancer imaging and therapy.

## Figures and Tables

**Table 1:** Photo-physical/photo-chemical data of compounds **6** and **7** in different solvents.

Solvent	Compounds	$\lambda_{max}^{abs}$ (nm)	${\lambda_{max}^{em}}^{a}$ (nm)	Φ_F_^b^	Stokes Shifts (nm)
DMSO	**6**	632	664	0.17	32
	**7**	707	755	0.067	48
PBS (0.05% Tween)	**6**	647	678	0.13	31
	**7**	705	739	0.042	34
DMSO:PBS = 1:4	**6**	652	696	0.089	44
	**7**	723	753	0.011	30

^a^ Excited at 610 nm. ^b^ Using unsubstituted zinc (II) phthalocyanine (ZnPc) in *N*,*N*′-dimethylformamide (DMF) as the standard (Φ_F_ = 0.28); Φ_F_ is the fluorescence quantum yield.

## Supplementary Materials

The supplementary materials including HRMS, ^1^H- and ^13^C-NMR spectra for compounds **4**–**7** are available online.
