# Supplementary material for: A H2O2-Responsive Boron Dipyrromethene-Based Photosensitizer for Imaging-Guided Photodynamic Therapy
Source: Molecules. 2018 Dec 21;24(1):32. doi: 10.3390/molecules24010032 (PMC6337283; doi:10.3390/molecules24010032)
Supplement: Supplementary file 1 [file molecules-24-00032-s001.pdf]

Electronic Supplementary Information

A H_2O_2-Responsive Boron Dipyrromethene-Based Photosensitizer for Imaging-Guided Photodynamic Therapy

Zhi-Wei Wang †, Dan Su †, Xiao-Qiang Li, Jing-Jing Cao, De-Chao Yang and Jian-Yong Liu *

Table of Contents

Figure S1. ^{1}H NMR spectrum of compound **4** in $CDCl_3$
Figure S2. ^{13}C NMR spectrum of compound **4** in $CDCl_3$
Figure S3. HRMS of compound **4**
Figure S4. ^{1}H NMR spectrum of compound **5** in $CDCl_3$
Figure S5. ^{13}C NMR spectrum of compound **5** in $CDCl_3$
Figure S6. HRMS of compound **5**
Figure S7. ^{1}H NMR spectrum of compound **6** in $CDCl_3$
Figure S8. ^{13}C NMR spectrum of compound **6** in MeOD
Figure S9. HRMS of compound **6**
Figure S10. ^{1}H NMR spectrum of compound **7** in $CDCl_3$
Figure S11. ^{13}C NMR spectrum of compound **7** in $CDCl_3$
Figure S12. HRMS of compound **7**

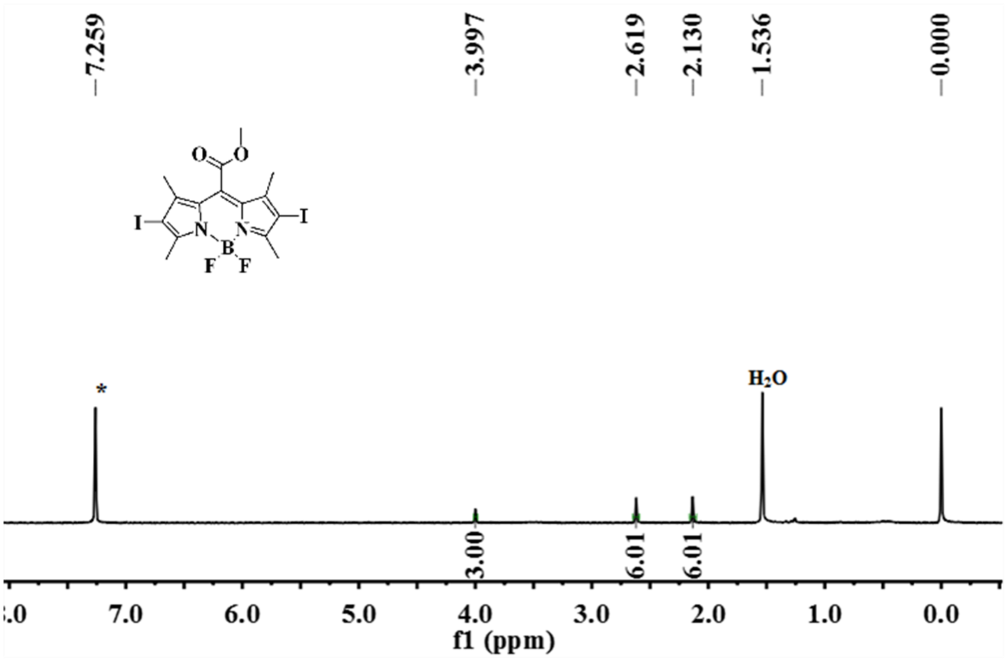

Figure S1. ^{1}H NMR spectrum of compound **4** in $CDCl_3$

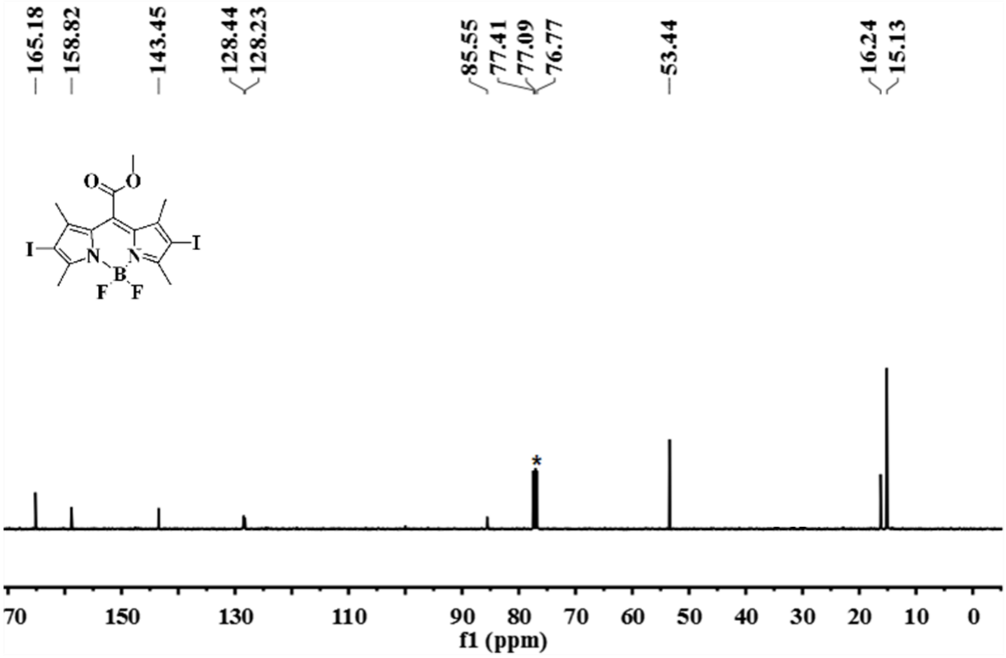

Figure S2. ^{13}C NMR spectrum of compound **4** in $CDCl_3$

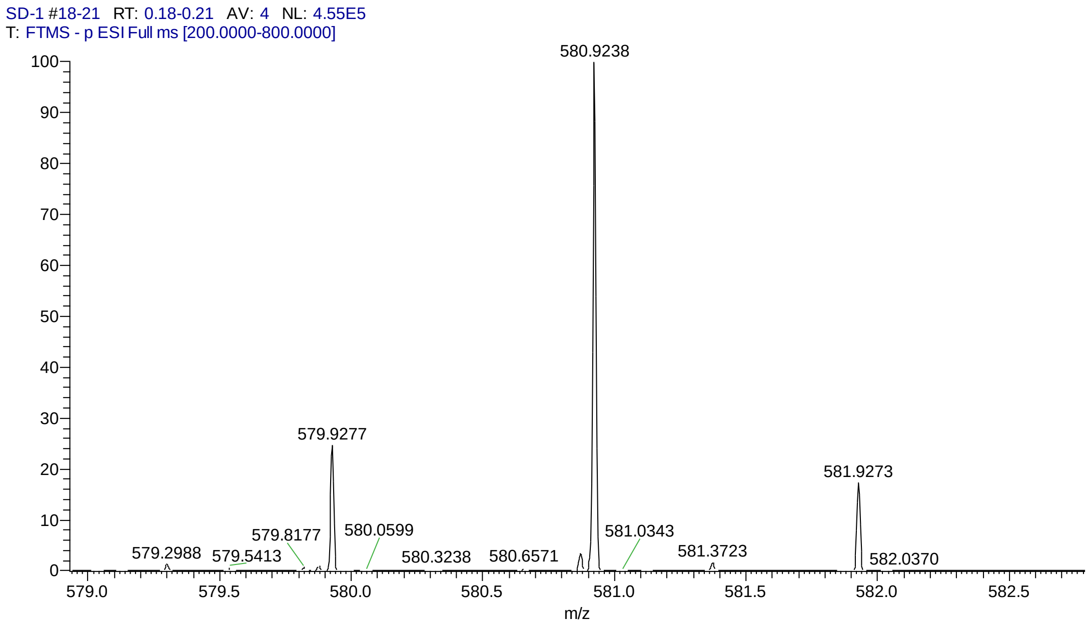

Figure S3. HRMS of compound **4**

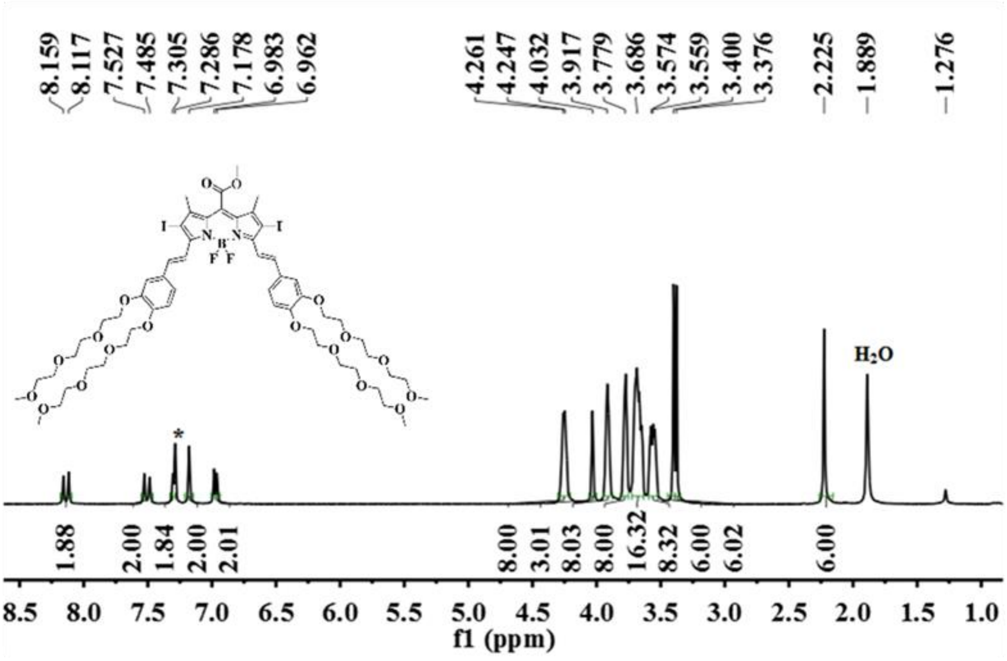

Figure S4. ^{1}H NMR spectrum of compound **5** in $CDCl_3$

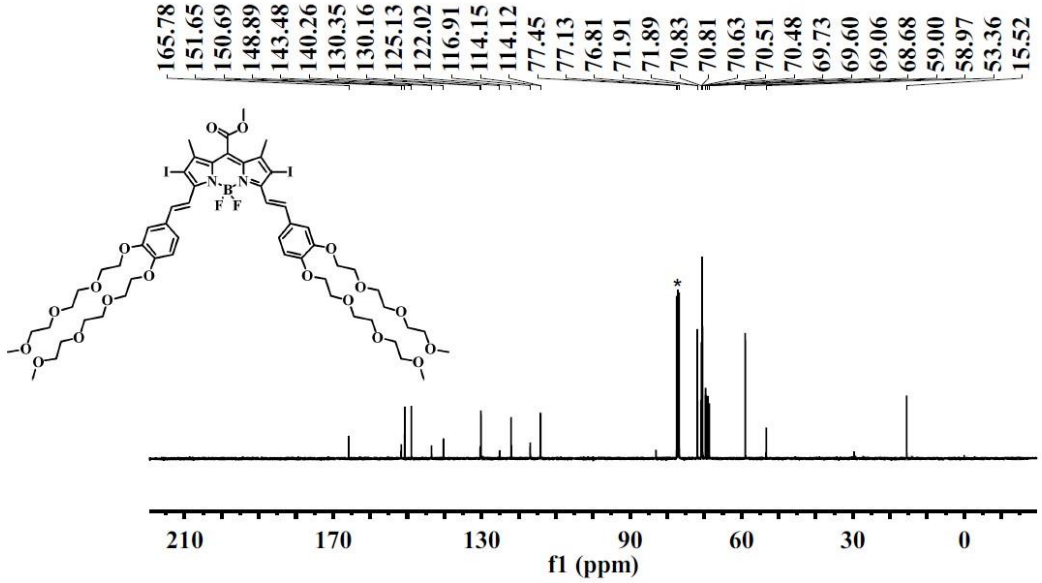

Figure S5. ^{13}C NMR spectrum of compound **5** in $CDCl_3$

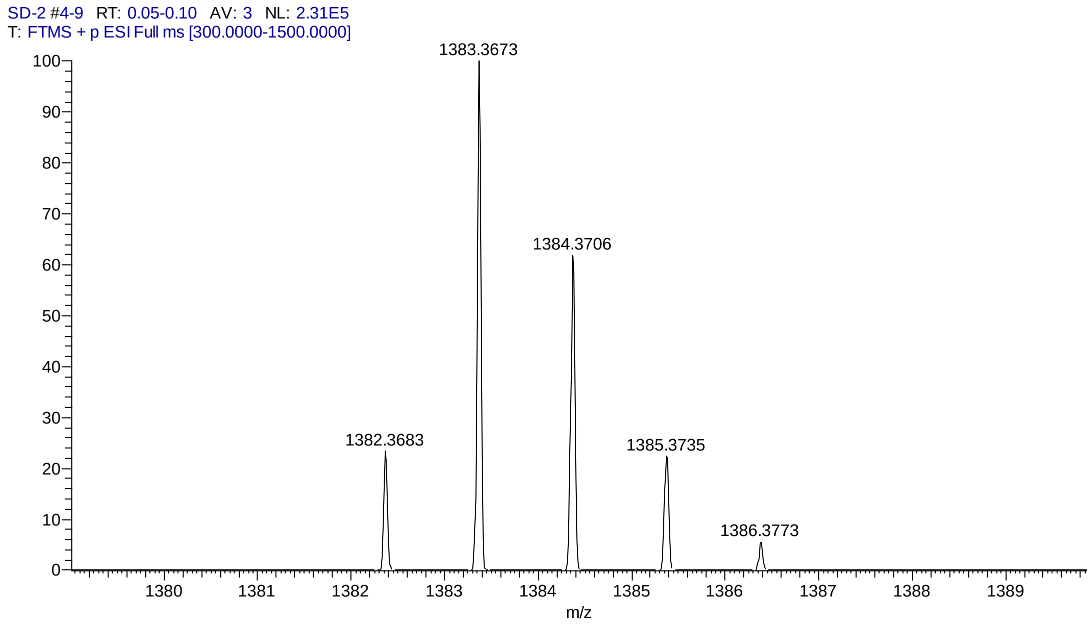

Figure S6. HRMS of compound **5**

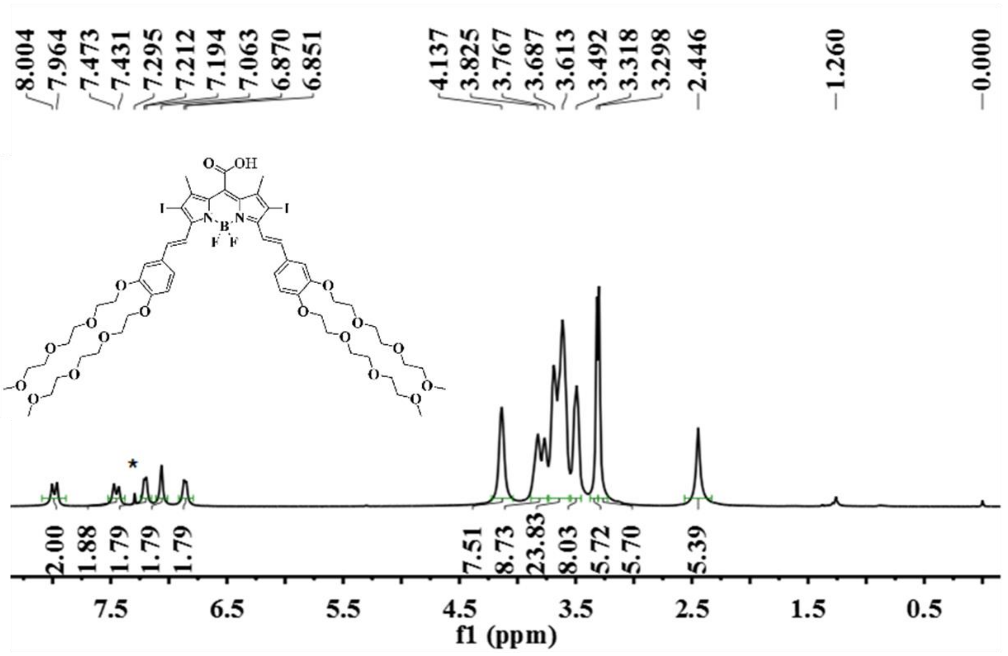

Figure S7. ^{1}H NMR spectrum of compound **6** in $CDCl_3$

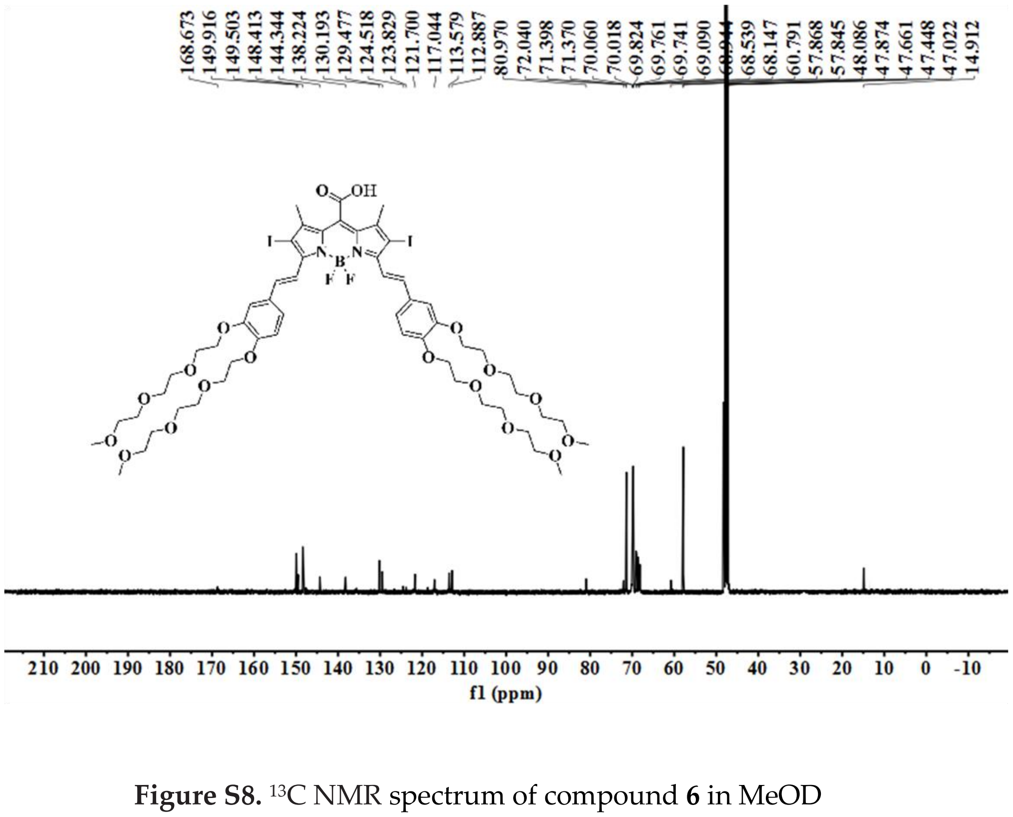

Figure S8. ^{13}C NMR spectrum of compound **6** in MeOD

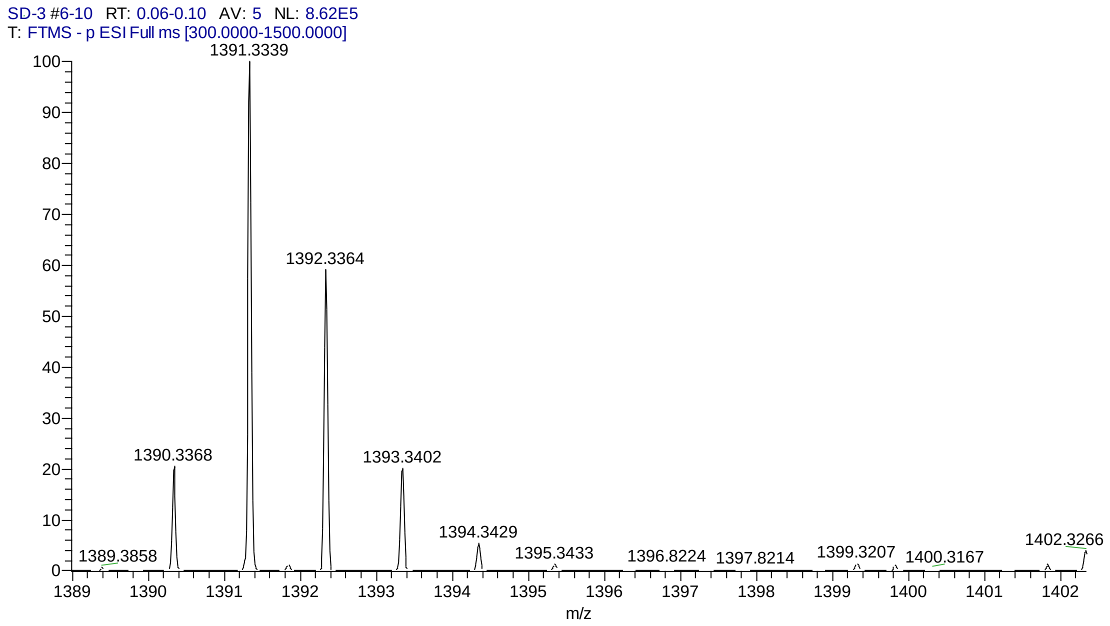

Figure S9. HRMS of compound **6**

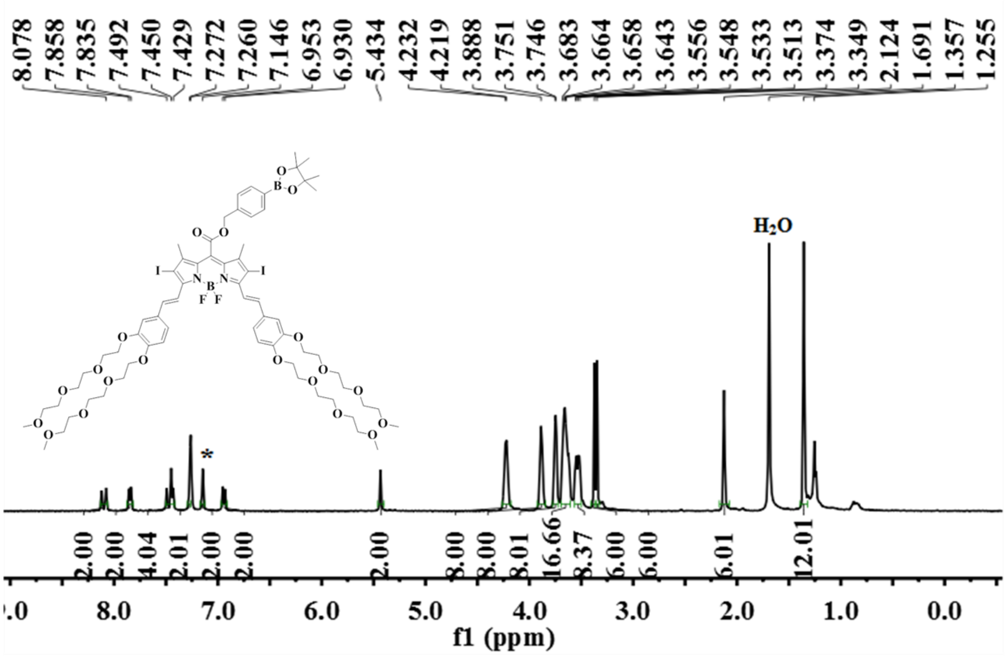

F**igure S10.** 1H NMR spectrum of compound **7** in $CDCl_3$.

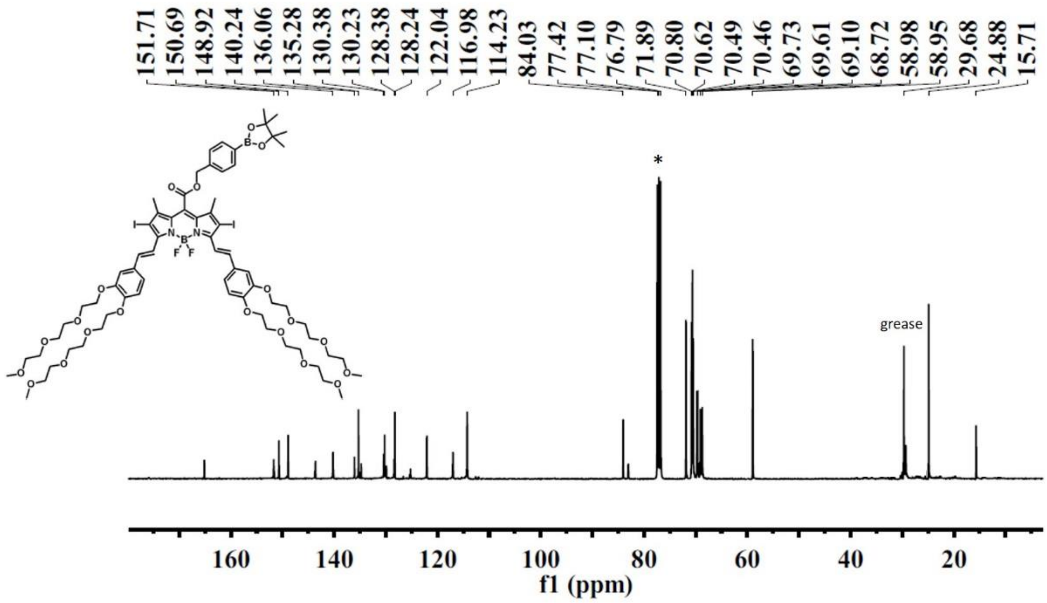

Figure S11. ^{13}C NMR spectrum of compound **7** in $CDCl_3$

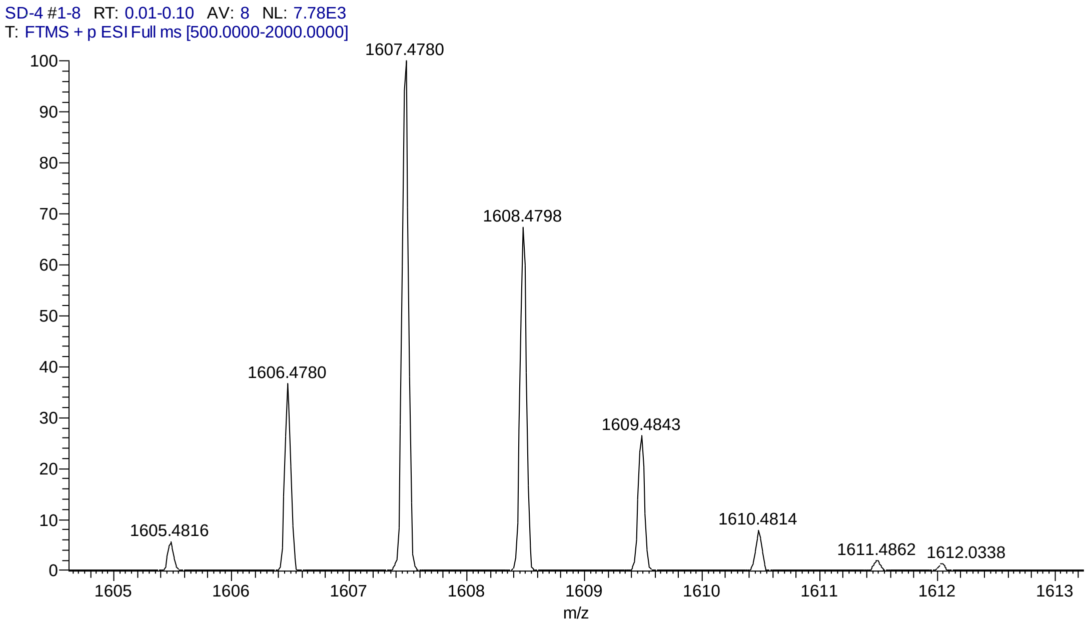

Figure S12. HRMS of compound **7**
